# Supplementary material for: Modulation of Serum Metabolic Profiles by Bifidobacterium breve BBr60 in Obesity: A Randomized Controlled Trial
Source: Foods. 2024 Nov 17;13(22):3655. doi: 10.3390/foods13223655 (PMC11594036; doi:10.3390/foods13223655)
Supplement: Supplementary file 1 [file foods-13-03655-s001.zip › foods-3284219-supplementary.pdf]

Table S1. Probiotics and placebo study populations: characteristics

Project	BBr60 (33)	Placebo (32)	*P* value
Woman	24 (72.7%)	19 (59.4%)	0.255
Man	9 (27.3%)	13 (40.6%)	
Age (year)	27.88±8.65	30.38±8.45	0.150
Weight (kg)	90.86 ± 10.45	93.56 ± 12.04	0.251
BMI (kg/m^2)	30.80 ± 3.21	31.96 ± 2.95	0.068
FP (%)	36.57 ± 6.77	38.91 ± 5.69	0.203
WHR (%)	0.99 ± 0.05	1.01 ± 0.05	0.500
FBG (mg/dL)	6.27±0.87	5.87±0.45	0.120
TC (mg/dL)	4.31±0.90	4.82±0.94	0.058
TG (mg/dL)	2.13±1.78	1.62±0.71	0.345
HDL-C (mg/dL)	1.15±0.27	1.29±0.31	0.484
LDL-C (mg/dL)	2.26±0.63	2.62±0.62	0.970
ALT (IU/L)	39.27±26.55	41.53±21.84	0.618
AST (IU/L)	61.18±58.70	49.56±17.88	0.969
TP (g/L)	74.45±9.70	72.47±3.46	0.787
ALB (g/L)	47.76±3.93	47.41±3.07	0.905
GLB (g/L)	26.70±7.28	25.06±3.05	0.697
A/G	1.90±0.41	1.92±0.29	0.273
TB (mg/dL)	13.58±6.58	17.38±14.83	0.453
BUN (mg/dL)	4.48±1.24	4.65±1.26	0.846
UA (mg/dL)	427.09±76.69	433.59±103.49	0.069
CRE (mg/dL)	76.48±14.80	71.16±15.77	0.543

Abbreviations: BMI, body mass index; BFP, body fat percentage; WHR, Waist-to-Hip Ratio; FBG, Fasting blood glucose; TC, Total cholesterol; TG, Triglyceride; HDL-C, high-density lipoprotein cholesterol; LDL-C, low-density lipoprotein cholesterol; ALT, Alanine aminotransferase; AST, Aspartate aminotransferase; TP, Total Protein; ALB, Albumin; GLB, Globular Proteins; A/G, Albumin/ Globulin; TB, Total bilirubin; BUN, Blood urea nitrogen; UA, Uric acid; CRE, Creatinine.

Table S2. Before and after 12-week intervention, clinic indicators for the BBr60 group and the placebo group

Variables	BBr60 (n=33)			Placebo (n=32)			*p*-Value
	Before (0 week)	After (12 week)	*p*-Value	Before (0 week)	After (12 week)	*p*-Value	
**Weight (kg)**	90.86±10.45	86.19±9.82	<0.0001	93.56±12.04	90.74±12.77	0.0006	0.114
**BMI (kg/m²)**	30.80±3.21	29.32±3.63	<0.0001	31.96±2.95	31.03±3.49	0.0019	0.057
**BFP (%)**	36.57±6.77	34.54±7.50	<0.0001	38.91±5.69	37.11±6.70	0.0003	0.150
**WHR (%)**	0.99±0.05	0.96±0.04	<0.0001	1.01±0.05	0.98±0.05	<0.0001	0.149
**FBG, mg/dL**	5.87±0.45	5.26±0.57	<0.0001	6.27±0.87	5.69±0.86	<0.0001	0.0381
**TC, mg/dL**	4.31±0.90	4.38±0.75	0.618	4.82±0.94	4.55±0.90	0.0058	0.4181
**TG, mg/dL**	2.13±1.78	1.98±1.12	0.8566	1.62±0.71	2.09±1.34	0.0984	0.9870
**HDL-C, mg/dL**	1.15±0.27	1.45±0.28	<0.0001	1.29±0.31	1.47±0.26	0.0071	0.7589
**LDL-C, mg/dL**	2.26±0.63	1.44±0.52	<0.0001	2.62±0.62	1.57±0.53	<0.0001	0.3483
**TP, g/L**	74.45±9.70	72.85±3.70	0.601	72.47±3.46	73.19±3.39	0.404	0.701
**ALB, g/L**	47.76±3.93	50.09±3.18	0.0003	47.41±3.07	49.31±2.01	0.0058	0.2037
**GLB, g/L**	26.70±7.28	22.76±3.85	0.0089	25.06±3.05	23.94±2.96	0.0499	0.170
**ALT, IU/L**	39.27±26.55	26.03±17.13	<0.0001	41.53±21.84	27.22±15.69	<0.0001	0.631
**AST, IU/L**	61.18±58.70	38.27±17.75	0.0002	49.56±17.88	39.59±17.49	0.0089	0.636
**BUN, mg/dL**	4.48±1.24	4.19±0.83	0.219	4.65±1.26	4.73±1.24	0.418	0.093

BMI, body mass index; WHR, waist hip ratio; BFR, body fat rate; FBG, fasting blood glucose; TG, total triglycerides; TC, total cholesterol, HDL-C, high-density lipoprotein cholesterol; LDL-C, low-density lipoprotein cholesterol; ALT, alanine aminotransferase; AST, aspartate aminotransferase; ALP, alkaline phosphatase; TP, total protein; ALB, Albumin, GLB, globular proteins; BUN, blood urea nitrogen.

Table S3. A comparison of serum metabolites between the BBr60 and placebo groups after 12 weeks of treatment

Name	MS2 score	mz	type	Formula	HMDB	CAS	KEGG ID	MEAN Placebo	MEAN BBr60	VIP	P-VALUE	FOLD CHANGE
2-Aminoheptanoic acid	3.99	146.1176	POS	C7H15NO2	HMDB0094649	44902-02-5		0.010775767	0.008938972	2.41659047	0.046209847	0.829543905
Methylimidazoleacetic acid	3.97	141.0659	POS	C6H8N2O2	HMDB0002820	2625-49-2	C05828	0.038324195	0.028595066	2.592091215	0.044579003	0.7461361
Proline	3.97	116.0705	POS	C5H9NO2	HMDB0000162	147-85-3	C00148	3.647489795	3.197717099	1.817375836	0.039970727	0.8766898
Hygric acid	3.96	128.0719	NEG	C6H11NO2	HMDB0094696	475-11-6	C22223	0.160897326	0.121545557	1.603037743	0.034451124	0.755423102
Taurochenodeoxycholic acid	3.96	498.29	NEG	C26H45NO6S	HMDB0000951	516-35-8	C05465	1.059504689	0.588903688	1.575693284	0.042996232	0.555829242
Glucuronic acid	3.95	193.0356	NEG	C6H10O7	HMDB0000127	528-16-5| 6556-12-3| 576-37-4| 72121-88-1	C00191	0.097384598	0.082692729	2.896341726	0.014247433	0.849135595
5-Methyl DL-glutamate	3.95	162.0761	POS	C6H11NO4	HMDB0061715	14487-45-7		0.035464306	0.024293132	3.718664725	6.79309E-06	0.685002329
Nordeoxycholic acid	3.95	377.2704	NEG	C23H38O4	HMDB0304947	53608-86-9		0.06959386	0.058960716	1.928744015	0.04962	0.847211461
Indole-3-propionic acid	3.94	188.0717	NEG	C11H11NO2	HMDB0002302	830-96-6	C22236	0.52071349	0.249768396	2.032007381	0.02633196	0.479665692
2-Hydroxyoctanoic acid	3.93	159.1028	NEG	C8H16O3	HMDB0000711	617-73-2		0.749645494	0.56498151	1.706462929	0.015511043	0.753664917
Citraconic acid	3.93	129.0196	NEG	C5H6O4	HMDB0000634	498-23-7	C02226	0.452012189	0.303729516	1.502726871	0.03074492	0.671949834
Azelaic acid	3.92	187.0979	NEG	C9H16O4	HMDB0000784	123-99-9	C08261	0.097070828	0.055386007	2.789894861	0.008773903	0.570573143
LPC(17:0/0:0)	3.92	510.3555	POS	C25H52NO7P	HMDB0012108	50930-23-9		0.519612673	0.604403853	1.365214854	0.004313019	1.163181509
1-Methylpseudouridine	3.92	257.0782	NEG	C10H14N2O6		13860-38-3		0.063712656	0.038931402	2.153425159	0.00353378	0.611046598
Undecanoic acid	3.92	185.1549	NEG	C11H22O2	HMDB0000947	112-37-8	C17715	0.251723511	0.201934888	1.549696391	0.027381352	0.802209089
Taurohyodeoxycholic acid	3.91	498.29	NEG	C26H45NO6S	HMDB0247202	386523		1.059504689	0.588903688	1.575693284	0.042996232	0.555829242
Propargylglycine	3.9	114.055	POS	C5H7NO2	HMDB0251529	198774-27-5		0.00682975	0.004970669	2.23545211	0.039970727	0.727796684
LPC(16:0)	3.9	496.3392	POS	C24H50NO7P	HMDB0010382	17364-16-8		45.56547328	50.47190457	1.684794447	0.001610287	1.107678708
Tauroursodeoxycholic acid	3.89	498.29	NEG	C26H45NO6S	HMDB0000874	14605-22-2	C16868	1.059504689	0.588903688	1.575693284	0.042996232	0.555829242

1-O-Hexadecyl-sn-glycero-3-phosphocholine (LPC(O-16:0/0:0))	3.88	482.3602	POS	C24H52NO6P	HMDB0243890	52691-62-0	C13903	0.773363713	0.972050194	2.446724081	5.83293E-05	1.256912081
PC(P-16:0/0:0)	3.88	480.3449	POS	C24H50NO6P	HMDB0010407	97802-53-4		0.75966693	0.878121352	1.317518406	0.013649833	1.155929419
Arabinono-1,4-lactone	3.87	147.03	NEG	C5H8O5	HMDB0001900	51532-86-6	C01114	0.282201187	0.213772578	1.03876749	0.023386606	0.757518354
Lauramine oxide	3.83	230.2477	POS	C14H31NO		1643-20-5		0.266232242	0.34949125	2.934776316	1.5368E-08	1.312730745
Lumichrome	3.83	241.0722	NEG	C12H10N4O2	HMDB0254199	1086-80-2	C01727	0.263619843	0.192819018	2.405921442	0.029586914	0.731428316
Taurodeoxycholic acid	3.81	498.29	NEG	C26H45NO6S	HMDB0000896	516-50-7	C05463	1.059504689	0.588903688	1.575693284	0.042996232	0.555829242
4-Isopropylbenzoic acid	3.78	163.0766	NEG	C10H12O2	HMDB0035268	536-66-3	C06578	0.081567645	0.054634784	1.614186475	0.016178446	0.669809502
Xanthoxylin	3.77	195.0663	NEG	C10H12O4	HMDB0029645	90-24-4	C10726	0.185143977	0.071306332	1.792050733	0.019102283	0.385139898
Indole-3-methyl acetate	3.76	188.0717	NEG	C11H11NO2	HMDB0029738	1912-33-0	C20635	0.52071349	0.249768396	2.032007381	0.02633196	0.479665692
5-Oxooctanoic acid	3.76	157.0871	NEG	C8H14O3		3637-14-7		0.437256845	0.324807913	2.490481328	0.000542637	0.742830938
Docebenone	3.75	325.1846	NEG	C21H26O3	HMDB0247732	80809-81-0	C01349	17.51540931	14.21098518	1.693029726	0.044579003	0.811341883
Pi-Methylimidazoleacetic acid (hydrochloride)	3.74	141.0659	POS	C6H8N2O2	HMDB0004988	4200-48-0		0.009811959	0.012152902	1.828515145	0.018331699	1.2385806
Phosphate	3.73	96.9698	NEG	H3O4P	HMDB0001429	7664-38-2	C00009	0.933985392	1.196161136	2.057372311	0.029586914	1.280706472
(1-Hydroxycyclohexyl)acetic acid	3.72	157.0871	NEG	C8H14O3		14399-63-4		0.437256845	0.324807913	2.490481328	0.000542637	0.742830938
Pyruvaldehyde	3.72	71.014	NEG	C3H4O2	HMDB0001167	78-98-8	C00546	2.638008323	2.13292159	1.787478887	0.010043807	0.808534822
Palmitoylcarnitine (Car(16:0))	3.72	400.3421	POS	C23H45NO4	HMDB0000222	2364-67-2	C02990	0.342362694	0.390036279	1.482340316	0.006969004	1.139248771
Royal jelly acid	3.72	185.1184	NEG	C10H18O3	HMDB0244269	14113-05-4		1.245952712	0.837577263	2.489675653	0.003359807	0.672238405
5-Hydroxyvalproic acid	3.69	159.1028	NEG	C8H16O3	HMDB0013898	53660-23-4	C16650	0.749645494	0.56498151	1.706462929	0.015511043	0.753664917
Dihexadecyldimethylammonium cation	2.75	494.5659	POS	C34H72N				0.007900872	0.01036268	2.249920551	7.73944E-05	1.311586912
PC(42:4)	2.74	848.6523	POS	C50H92NO8P				0.170364202	0.181345521	1.32815883	0.047889824	1.064457903
Glycerophospho-N-palmitoylethanolamine	2.74	454.2931	POS	C21H44NO7P				0.184992272	0.226161176	2.278573348	0.000376278	1.222543916
1-O-Hexadecyl-2-O-(2E-butenoyl)-sn-glyceryl-3-phosphocholine	2.74	550.3865	POS	C28H56NO7P				0.178614422	0.154726708	1.126481089	0.039970727	0.866260999
2-(5-Oxovaleryl)phosphatidylcholine	2.74	594.3761	POS	C29H56NO9P				0.026237182	0.015609368	2.460811642	0.000143255	0.594933086
1-Isobutyl-1H-pyrazol-5-ylamine	2.74	140.1182	POS	C7H13N3				0.037982919	0.038128421	1.59008959	0.012519673	1.003830702
Tetraethylammonium cation	2.74	130.159	POS	C8H20N				0.013440446	0.019216229	1.30967824	0.002882948	1.429731471
Tetrapropylammonium cation	2.74	186.2215	POS	C12H28N				0.017354866	0.025145995	3.245205584	9.53583E-10	1.44893057

Tetradecylamine	2.74	214.2529	POS	C14H31N	HMDB0258887			0.003670279	0.005260101	3.055389508	6.26364E-05	1.433160938
1-Hexadecylamine	2.74	242.2841	POS	C16H35N	HMDB0243891			0.058741561	0.07831042	2.884383698	3.46279E-06	1.333134818
3-Cyclohexyl-2-hydroxypropanoic acid	2.74	171.1028	NEG	C9H16O3				1.939928972	1.367244391	1.912716474	0.004757327	0.704790954
11-Hydroxyundecanoic acid	2.74	201.1497	NEG	C11H22O3				0.093980189	0.07190703	1.392729103	0.04962	0.765129658
Tetraoctylammonium cation	2.74	466.5343	POS	C32H68N				0.004672194	0.006462613	2.672913146	9.41585E-06	1.383207299
6-Ethoxy-4-methylcoumarin	2.74	205.0858	POS	C12H12O3				0.001136757	0.000936682	1.670977931	0.034451124	0.823995299
Tetradecenedioylcarnitine (Car(14:1-O2))	2.73	400.2696	POS	C21H37NO6	HMDB0241367			0.002910034	0.002417229	2.181453962	0.029586914	0.830653126
1-O-Octadecyl-sn-glyceryl-3-phosphorylcholine	2.73	510.3914	POS	C26H56NO6P	HMDB0011149	74430-89-0	C04317	0.086220324	0.114099524	2.323932604	0.000186677	1.323348357
Carbaprostacyclin	2.73	349.2386	NEG	C21H34O4				0.087327898	0.072620496	1.388660541	0.018331699	0.83158415
(3-Methylbutyl)(6-methylheptan-2-yl)amine	2.73	200.2371	POS	C13H29N				0.003419869	0.00473306	2.581638431	4.47313E-06	1.383988646
1-Hexadecyl-2-(5Z,8Z,11Z,14Z-eicosatetraenoyl)-sn-glycero-3-phosphocholine	2.73	768.5891	POS	C44H82NO7P				1.642188647	1.868230019	1.486244106	0.024335466	1.13764641
1-O-Hexadecyl-2-O-(5Z,8Z,11Z,14Z,17Z-eicosapentaenoyl)-sn-glyceryl-3-phosphorylcholine	2.73	766.5738	POS	C44H80NO7P	HMDB0039528	132196-28-2		1.575432096	1.762440684	1.317637883	0.046209847	1.118703046
2-(3-Methylbutoxy)acetic acid	2.73	145.0871	NEG	C7H14O3				0.211887622	0.157482715	2.361314926	0.010501495	0.743236974
4-(1-Pyrazolyl)benzaldehyde	2.73	173.0707	POS	C10H8N2O				0.084086765	0.103887458	1.637059451	0.012519673	1.23547931
4-Ethoxy-4-oxobut-2-enoic acid	2.73	143.0351	NEG	C6H8O4	HMDB0246416			1.723029682	1.438874905	1.756023331	0.031940989	0.835084224
Mupirocin	2.73	499.2917	NEG	C26H44O9	HMDB0014554	12650-69-0	C11758	0.190537607	0.149014842	1.410359165	0.010977327	0.782075752
1-Octadecylamine	2.73	270.3153	POS	C18H39N	HMDB0029586	124-30-1		0.08069608	0.106721625	2.326447671	7.49152E-07	1.322513125
DIHYDROJASMONIC_ACID	2.73	211.1342	NEG	C12H20O3	HMDB0033601	98674-52-3		1.142512907	0.957764209	1.498933417	0.02633196	0.838296183
Hexenoylcarnitine (Car(6:1))	2.72	258.17	POS	C13H23NO4	HMDB0013161			0.024084473	0.015757213	2.923513421	0.000376278	0.654247755
8-(Trifluoromethyl)quinolin-4-ol	2.72	212.0332	NEG	C10H6F3NO				0.077048528	0.0585106	2.51015692	0.020727325	0.759399315
Hexadecanedioic acid, 3,3,14,14-tetramethyl-	2.72	341.27	NEG	C20H38O4	HMDB0254402			0.165751796	0.12172558	2.044147056	0.028466032	0.734384682
8-Methoxy-4-oxo-1,4-dihydroquinoline-2-carboxylic acid	2.72	218.0459	NEG	C11H9NO4	HMDB0060426		C05830	0.093374736	0.070459632	1.155671893	0.008773903	0.754589896
1,2-dihydroxyheptadec-16-en-4-yl acetate	2.72	327.2543	NEG	C19H36O4	HMDB0031045	59499-18-2		0.105446057	0.064755026	1.990240093	0.046209847	0.614105709
1-Oleoyl-2-palmitoyl-sn-glycero-3-phosphocholine	2.71	760.5832	POS	C42H82NO8P	HMDB0008100			0.259058899	0.345287208	2.290196497	0.031940989	1.332852144
3-Dodecyloxypropylamine	2.71	244.2632	POS	C15H33NO				0.022028618	0.025027567	1.953865834	0.000480894	1.136138752
(3E,5E)-2-(4-Methoxy-4-oxobutyl)nona-3,5-dienoic acid	2.71	253.1447	NEG	C14H22O4				0.122524504	0.084206379	2.165320613	0.002221643	0.687261541

2-Oxiraneoctanoic acid, .eta.-hydroxy-3-octyl-	2.71	313.2388	NEG	C18H34O4			0.272160144	0.142976287	2.11436582	0.002738055	0.525338814
PC(33:1)	2.7	728.5569	POS	C41H80NO8P			0.037792115	0.030396775	1.474684284	0.042996232	0.804315272
N-Methyldioctylamine	2.7	256.2998	POS	C17H37N			0.001609692	0.002502867	2.308202421	0.000332215	1.554873467
Nonadienoylcarnitine (Car(9:2))	2.7	298.2015	POS	C16H27NO4	HMDB0241768		0.005981321	0.004392406	1.252350541	0.012519673	0.734353773
4-(4-Amino-3-nitrophenyl)-1(2H)-phthalazinone	2.69	283.0835	POS	C14H10N4O3			0.012797076	0.007761474	1.8574627	0.035767125	0.606503722
2-(Naphthalen-1-yl)-1H-1,3-benzodiazole	2.69	245.1065	POS	C17H12N2			0.069865304	0.040276854	1.650183712	0.044579003	0.576492925
cis-4-Hydroxycyclohexanecarboxylic acid	2.69	143.0715	NEG	C7H12O3			0.114721202	0.090933739	1.674254681	0.010501495	0.79264981
5-Isopropyl-5-methylhydantoin	2.68	157.0971	POS	C7H12N2O2			0.01663754	0.010304304	2.97061802	0.000920541	0.619340566
LPC(O-20:0)	2.68	538.4245	POS	C28H60NO6P			0.014155105	0.018045617	2.056349982	0.000920541	1.274848637
Decenedioylcarnitine (Car(10:1-O2))	2.68	326.1963	POS	C17H29NO6	HMDB0241084		0.007810764	0.005341184	1.74751257	0.010977327	0.683823486
4-Nitro-1,3-benzenediamine	2.67	154.0611	POS	C6H7N3O2			0.013720251	0.011264446	1.711148133	0.034451124	0.821008766
Decatetraenoylcarnitine (Car(10:4))	2.67	308.1847	POS	C17H25NO4			0.041408507	0.024445721	1.839083327	0.016870533	0.590355057
LPC(19:0)	2.65	538.3861	POS	C27H56NO7P		108273-88-7	0.065712087	0.078749501	1.913343176	0.006344396	1.198402075
N6-(1-Iminoethyl)-L-lysine	2.64	188.1394	POS	C8H17N3O2	HMDB0249982		0.027489433	0.016110085	2.798895674	0.003715766	0.586046476
(2E)-4-Hydroxybut-2-enoic acid	2.63	101.0245	NEG	C4H6O3	HMDB0003381	24587-49-3	2.77593817	2.183413718	1.386017879	0.04962	0.786549838
6-(1-Pyrrolidinyl)-1H-purine	2.63	188.0929	NEG	C9H11N5			0.091064897	0.044763694	2.738858599	0.000257973	0.491558163
Heptadecanoyl_carnitine	2.63	414.3578	POS	C24H47NO4	HMDB0006210	106182-29-0	0.008436475	0.009830305	1.482932739	0.042996232	1.165214792
Auxin_b	2.62	311.2216	POS	C18H30O4	HMDB0038484		0.004766989	0.009467785	1.100169373	0.001155307	1.986114536
PC(42:7)	2.62	860.6098	POS	C50H86NO8P			0.024971337	0.028392896	1.323763325	0.003906082	1.137019478
Damnacanthal	2.61	283.0624	POS	C16H10O5	HMDB0250841		0.038298658	0.02230442	1.89512341	0.027381352	0.582381255
(Diethylamino)(oxo)acetic acid	2.61	146.0812	POS	C6H11NO3			0.176940219	0.070981688	2.961619913	0.001221924	0.401161974
Triptophenolide	2.55	311.1689	NEG	C20H24O3			19.28114119	15.50615561	1.723120737	0.034451124	0.804213581
8-Geranyl-7-hydroxycoumarin	2.55	297.1532	NEG	C19H22O3			7.479271766	5.836066258	1.936189901	0.035767125	0.78029873
sec-Butyltriphenylphosphonium cation	2.53	319.1653	POS	C22H24P			0.016608916	0.02626612	1.443864581	0.012519673	1.581447004
5-(4-Hydroxybenzylidene)-1,3-dimethyl-2,4,6(1H,3H,5H)-pyrimidinetrione	2.53	259.0757	NEG	C13H12N2O4			0.024924614	0.006692952	2.28824124	0.000376278	0.268527817
Gln-Arg	2.53	301.1592	NEG	C11H22N6O4			0.179203713	0.121116465	1.509976308	0.004105054	0.675859128

5-Amino-2-(5-amino-1,3-benzoxazol-2-yl)phenol	2.53	240.075	NEG	C13H11N3O2				0.133858572	0.070131112	2.548747018	0.001443221	0.523919474
rhein	2.52	285.0426	POS	C15H8O6	HMDB0032876	478-43-3	C10401	0.002175821	0.001589819	2.08014585	0.004105054	0.730675491
1-(4-Chlorophenyl)-2-[(5-methyl-4H-1,2,4-triazol-3-yl)sulfanyl]ethanone	2.51	266.0179	NEG	C11H10ClN3OS				33.97031374	24.36731745	1.70178842	0.047889824	0.717312111
Tetrahydromagnolol	2.5	269.1507	NEG	C18H22O2				0.372046474	0.286642072	1.277632902	0.021583373	0.770446952
1-O-Hexadecyl-2-O-(N-methylcarbamoyl)-sn-glyceryl-3-phosphorylcholine	2.5	539.39	POS	C26H55N2O7P				0.018973418	0.022309515	1.542075246	0.013649833	1.175830003
(R)-1-O-b-D-glucopyranosyl-1,3-octanediol	2.47	309.1887	POS	C14H28O7	HMDB0029362	120727-21-1		0.006704524	0.003832675	1.931955117	0.017588036	0.571655035
Oxfendazole	2.44	314.0558	NEG	C15H13N3O3S	HMDB0031812	53716-50-0		0.040883191	0.023490001	2.137689255	0.010043807	0.57456378
PC(18:1(9Z)/18:3(9Z,12Z,15Z))	2.39	782.5693	POS	C44H80NO8P	HMDB0008074			0.133689946	0.16781916	2.385612762	0.023386606	1.255286314
9,10-Dihydrojasmonic acid	2.37	211.1341	NEG	C12H20O3				0.225963294	0.175886908	1.38163478	0.047889824	0.778387077
PC(20:2(11Z,14Z)/18:1(9Z))	2.37	812.6161	POS	C46H86NO8P	HMDB0008334			0.026165337	0.082012572	2.370707471	0.029586914	3.134397766
3-[5-(2-Methylpropyl)-3,6-dioxopiperazin-2-yl]propanoic acid	2.35	243.134	POS	C11H18N2O4				0.020958186	0.012905695	1.629956806	0.010977327	0.615783008
PC(18:3(6Z,9Z,12Z)/15:0)	2.35	742.5372	POS	C41H76NO8P	HMDB0008165			0.212899606	0.162788683	1.031505005	0.00730121	0.764626511
PC(14:0/P-18:1(9Z))	2.35	716.5566	POS	C40H78NO7P	HMDB0007898		C00157	0.016664768	0.020108578	1.557503399	0.010501495	1.206652163
PC(22:4(7Z,10Z,13Z,16Z)/P-18:0)	2.34	822.636	POS	C48H88NO7P	HMDB0008653			0.270470143	0.281873971	1.334588339	0.034451124	1.042162982
Tuberonic acid	2.28	227.1281	POS	C12H18O4				0.004085105	0.002841078	2.809306597	0.011471895	0.695472448
4-Oxo-4-((3-oxodecan-2-yl)amino)butanoic acid	2.26	272.1857	POS	C14H25NO4				0.028734764	0.018424516	2.883503292	0.00024197	0.641192536
3-Oxocyclobutanecarboxylic acid	2.25	113.0246	NEG	C5H6O3				2.610108977	2.151413909	1.495226759	0.047889824	0.824262101
Putative Phenylalanine conjugated chenodeoxycholic acid	2.25	540.3663	POS	C33H49NO5	HMDB0242391			0.033993296	0.039081831	1.127018634	0.004105054	1.149692314
13,14-dihydro-PGE1	2.24	357.2651	POS	C20H36O5	HMDB0002689	19313-28-1		0.002793579	0.001336792	1.525129069	0.028466032	0.478523198
Dye X-25377-20	2.23	307.048	NEG	C12H12N4O4S				0.05372165	0.034161583	2.522234681	0.007647311	0.635899737
Dimethyl 5-[(3-chloropropanoyl)amino]-3-methylthiophene-2,4-dicarboxylate	2.22	320.0347	POS	C12H14ClNO5S				0.002391449	0.001657976	2.728344267	0.028466032	0.693293367
(2-aminoethoxy)[2-[octadec-9-enoyloxy]-3-[octadeca-1.11-dien-1-yloxy]propoxy]phosphinic acid	2.22	726.5441	NEG	C41H78NO7P	HMDB0011408			5.622329711	3.952614794	1.167637504	0.044579003	0.703020811
SM_C16:1	2.22	716.5776	POS	C40H80N2O6P	HMDB0029216			0.057357359	0.062855404	1.658362068	0.027381352	1.095855953
Aurintricarboxylic acid	2.2	421.0604	NEG	C22H14O9	HMDB0248731			1.223687619	0.761650015	2.120086557	0.000576155	0.622421934
Fenhexamid	2.2	300.0576	NEG	C14H17Cl2NO2	HMDB0252201		C18593	0.082741004	0.068347549	1.66092983	0.038525925	0.826042054
5-(2-Methylbutan-2-yl)-4,5,6,7-tetrahydro-2H-indazole-3-carbohydrazide	2.17	251.1851	POS	C13H22N4O				0.053691581	0.039765148	1.79199485	0.029586914	0.740621658

3-Hydroxystigmast-5-en-7-one	2.17	429.3772	POS	C29H48O2	HMDB0034422	2034-74-4		0.027580423	0.038390615	2.609287033	0.000730021	1.391951632
5-Azacytidine	2.16	243.0753	NEG	C8H12N4O5				0.005328862	0.002941439	1.61077272	0.02531696	0.551982605
4,5-Epoxy-7Z,10Z,13Z,16Z,19Z-docosapentaenoic acid, methyl ester	2.16	359.2625	POS	C23H34O3				0.010715268	0.006943831	2.289898096	0.028466032	0.648031511
Gallomyricitrin	2.14	615.1033	NEG	C28H24O16				0.032246272	0.020937223	2.482663716	0.008773903	0.649291264
5-O-Methylsulochrin	2.07	345.0942	NEG	C18H18O7	HMDB0341175			0.059048918	0.049765649	2.279393039	0.035767125	0.842786801
(7E)-11-Hydroxy-4,5,8,13,13-pentamethyl-3-(2-methylpropyl)-3,3a,4,6a,9,10,11,11a,14a,15-decahydro-1H-[1,3]dioxolo[9,10]oxacyclododecino[2,3-d]isoindole-1,16(2H)-dione	2.06	476.3062	POS	C27H41NO6				0.012293059	0.007437848	2.267621147	0.04962	0.605044509
Sebacic acid	1.85	201.1133	NEG	C10H18O4	HMDB0000792	111-20-6	C08277	0.073158647	0.058497371	1.935329386	0.023386606	0.799596128
4-Chlorophenoxyacetate	1.74	202.0333	NEG	C8H7ClO3				0.009576606	0.014378786	1.481271779	0.021583373	1.501449046
M303T119	1.4	303.1733	POS	C17H24N2O4				0.010228351	0.005388993	1.889759628	0.003715766	0.526868212
(2S)-2-{[1-(R)-Carboxyethyl]amino}pentanoate	1.35	210.075	NEG	C8H15NO4				0.008655038	0.006163602	1.187526188	0.013074134	0.712140313
M577T46	1.33	577.3752	NEG	C33H54O8				0.496766941	0.463285677	1.080483097	0.033176069	0.932601665

Table S4. KEGG Enrichment data matrix

**Pathway**	**Description**	**Compounds(dem)**	**Percent**	**Rich_factor**	***p*_value**	**up_nums**	**down_nums**	**DA_score**
hsa01100	Metabolic pathways	C05828;C00148;C00191;C02226;C01114;C06578;C00546;C00157;C04317	60	0.002927781	0.84697259	2	7	-0.555555556
hsa00053	Ascorbate and aldarate metabolism	C00191;C01114	13.33333333	0.035087719	0.015126731	0	2	-1
hsa02010	ABC transporters	C00148;C00009	13.33333333	0.014492754	0.076637229	1	1	0
hsa04978	Mineral absorption	C00148;C00009	13.33333333	0.068965517	0.004062645	1	1	0
hsa01210	2-Oxocarboxylic acid metabolism	C02226	6.666666667	0.006944444	0.389053273	0	1	-1
hsa01212	Fatty acid metabolism	C02990	6.666666667	0.008196721	0.340589744	1	0	1
hsa01230	Biosynthesis of amino acids	C00148	6.666666667	0.0078125	0.354151188	0	1	-1
hsa01250	Biosynthesis of nucleotide sugars	C00191	6.666666667	0.005	0.497835205	0	1	-1

hsa01240	Biosynthesis of cofactors	C00191		6.666666667	0.00304878	0.682373235	0	1	-1
hsa00040	Pentose and glucuronate interconversions	C00191		6.666666667	0.016949153	0.181216336	0	1	-1
hsa00520	Amino sugar and nucleotide sugar metabolism	C00191		6.666666667	0.008474576	0.331401629	0	1	-1
hsa00620	Pyruvate metabolism	C00546		6.666666667	0.03125	0.102469951	0	1	-1
hsa00640	Propanoate metabolism	C00546		6.666666667	0.024390244	0.12947183	0	1	-1
hsa00562	Inositol phosphate metabolism	C00191		6.666666667	0.021276596	0.147049783	0	1	-1
hsa00190	Oxidative phosphorylation	C00009		6.666666667	0.083333333	0.039642339	1	0	1
hsa00071	Fatty acid degradation	C02990		6.666666667	0.02	0.155713996	1	0	1
hsa00120	Primary bile acid biosynthesis	C05465		6.666666667	0.021276596	0.147049783	0	1	-1
hsa00564	Glycerophospholipid metabolism	C00157		6.666666667	0.017857143	0.172796551	1	0	1
hsa00565	Ether lipid metabolism	C04317		6.666666667	0.04	0.080930226	1	0	1
hsa00590	Arachidonic acid metabolism	C00157		6.666666667	0.012658228	0.235336509	1	0	1
hsa00591	Linoleic acid metabolism	C00157		6.666666667	0.035714286	0.090219945	1	0	1
hsa00592	alpha-Linolenic acid metabolism	C00157		6.666666667	0.022727273	0.138302639	1	0	1
hsa00260	Glycine, serine and threonine metabolism	C00546		6.666666667	0.020833333	0.149947032	0	1	-1
hsa00290	Valine, leucine and isoleucine biosynthesis	C02226		6.666666667	0.043478261	0.074687977	0	1	-1
hsa00330	Arginine and proline metabolism	C00148		6.666666667	0.014492754	0.208708161	0	1	-1
hsa00340	Histidine metabolism	C05828		6.666666667	0.021276596	0.147049783	0	1	-1
hsa00380	Tryptophan metabolism	C05830		6.666666667	0.012048193	0.245751751	0	1	-1
hsa00470	D-Amino acid metabolism	C00148		6.666666667	0.014492754	0.208708161	0	1	-1
hsa00740	Riboflavin metabolism	C01727		6.666666667	0.041666667	0.077814031	0	1	-1
hsa00982	Drug metabolism - cytochrome P450	C16650		6.666666667	0.011494253	0.256034468	0	1	-1
hsa00970	Aminoacyl-tRNA biosynthesis	C00148		6.666666667	0.019230769	0.161444428	0	1	-1
hsa04928	Parathyroid hormone synthesis, secretion and action	C00009		6.666666667	0.090909091	0.036395694	1	0	1
hsa04976	Bile secretion	C05465		6.666666667	0.010309278	0.281171703	0	1	-1

hsa04974	Protein digestion and absorption	C00148		6.666666667	0.021276596	0.147049783	0	1	-1
hsa04979	Cholesterol metabolism	C05465		6.666666667	0.1	0.033138807	0	1	-1
hsa04723	Retrograde endocannabinoid signaling	C00157		6.666666667	0.052631579	0.062084589	1	0	1
hsa05230	Central carbon metabolism in cancer	C00148		6.666666667	0.027027027	0.117565987	0	1	-1
hsa05231	Choline metabolism in cancer	C00157		6.666666667	0.090909091	0.036395694	1	0	1
hsa05012	Parkinson disease	C00009		6.666666667	0.038461538	0.084036591	1	0	1
hsa05022	Pathways of neurodegeneration - multiple diseases	C00009		6.666666667	0.03125	0.102469951	1	0	1
hsa05415	Diabetic cardiomyopathy	C00546		6.666666667	0.025641026	0.123537777	0	1	-1

DA score, Differential Abundance Score

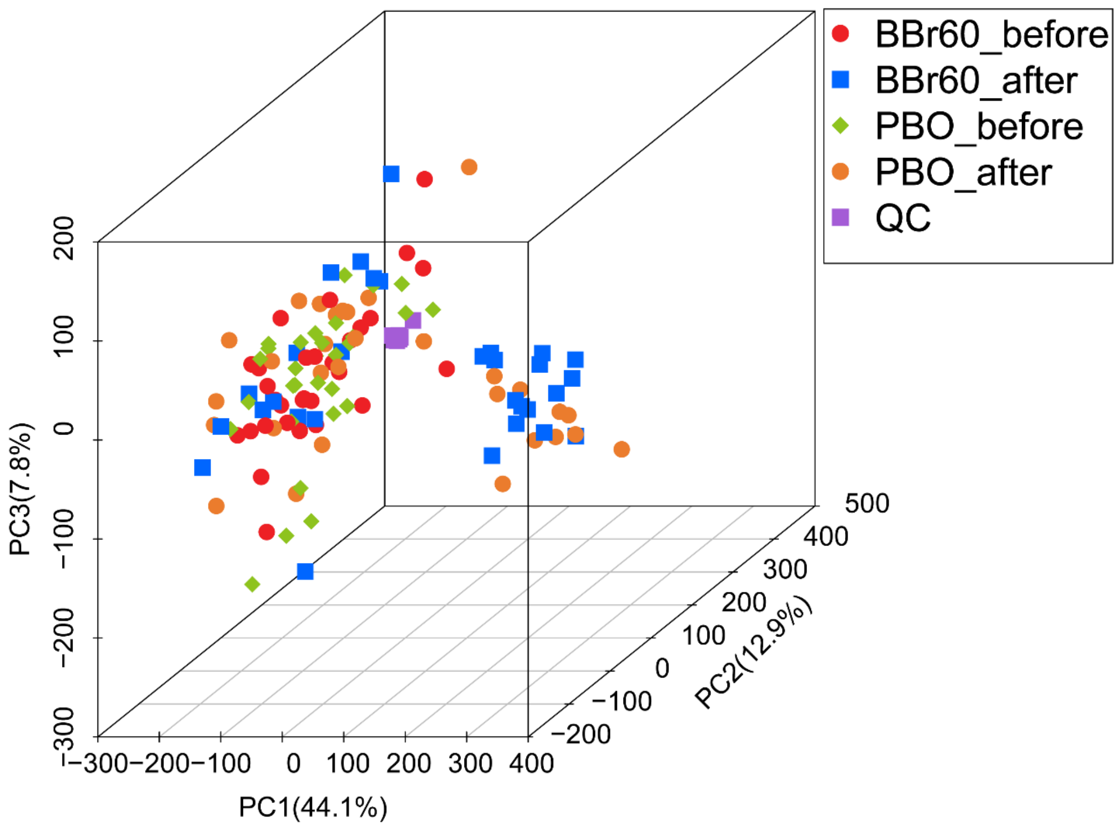

Figure S1. The serum metabolic profile's effect (scores plot of PCA) of BBr60 in adult population with overweight/obese in 0 and twelfth week.
